# The Application of Natural Killer Cell Immunotherapy for the Treatment of Cancer

**DOI:** 10.3389/fimmu.2015.00578

**Published:** 2015-11-17

**Authors:** Katayoun Rezvani, Rayne H. Rouce

**Affiliations:** ^1^Department of Stem Cell Transplantation, Division of Cancer Medicine, The University of Texas MD Anderson Cancer Center, Houston, TX, USA; ^2^Department of Pediatrics, Texas Children’s Cancer and Hematology Centers, Baylor College of Medicine, Houston, TX, USA; ^3^Center for Cell and Gene Therapy, Baylor College of Medicine Houston Methodist Hospital and Texas Children’s Hospital, Houston, TX, USA

**Keywords:** natural killer cells, adoptive immunotherapy, CAR NK cells, ADCC, anti-KIR antibody, NK-92, transplantation

## Abstract

Natural killer (NK) cells are essential components of the innate immune system and play a critical role in host immunity against cancer. Recent progress in our understanding of NK cell immunobiology has paved the way for novel NK cell-based therapeutic strategies for the treatment of cancer. In this review, we will focus on recent advances in the field of NK cell immunotherapy, including augmentation of antibody-dependent cellular cytotoxicity, manipulation of receptor-mediated activation, and adoptive immunotherapy with *ex vivo*-expanded, chimeric antigen receptor (CAR)-engineered, or engager-modified NK cells. In contrast to T lymphocytes, donor NK cells do not attack non-hematopoietic tissues, suggesting that an NK-mediated antitumor effect can be achieved in the absence of graft-vs.-host disease. Despite reports of clinical efficacy, a number of factors limit the application of NK cell immunotherapy for the treatment of cancer, such as the failure of infused NK cells to expand and persist *in vivo*. Therefore, efforts to enhance the therapeutic benefit of NK cell-based immunotherapy by developing strategies to manipulate the NK cell product, host factors, and tumor targets are the subject of intense research. In the preclinical setting, genetic engineering of NK cells to express CARs to redirect their antitumor specificity has shown significant promise. Given the short lifespan and potent cytolytic function of mature NK cells, they are attractive candidate effector cells to express CARs for adoptive immunotherapies. Another innovative approach to redirect NK cytotoxicity towards tumor cells is to create either bispecific or trispecific antibodies, thus augmenting cytotoxicity against tumor-associated antigens. These are exciting times for the study of NK cells; with recent advances in the field of NK cell biology and translational research, it is likely that NK cell immunotherapy will move to the forefront of cancer immunotherapy over the next few years.

## Introduction

Natural killer (NK) cell-mediated cytotoxicity contributes to the innate immune response against various malignancies, including leukemia (1, 2). The antitumor effect of NK cells is a subject of intense investigation in the field of cancer immunotherapy. In this review, we will focus on recent advances in NK cell immunotherapy, including augmentation of antibody-dependent cytotoxicity, manipulation of receptor-mediated activation, and adoptive immunotherapy with *ex vivo*-expanded, chimeric antigen receptor (CAR)-engineered, or engager-modified NK cells.

## Biology of NK Cells Relevant to Adoptive Immunotherapy

Natural killer cells are characterized by the lack of CD3/TCR molecules and by the expression of CD16 and CD56 surface antigens. Around 90% of circulating NK cells are CD56^dim^, characterized by their distinct ability to mediate cytotoxicity in response to target cell stimulation (3, 4). This subset includes the alloreactive NK cells that play a central role in targeting leukemia cells in the setting of allogeneic hematopoietic stem cell transplant (HSCT) (5). The remaining NK cells, predominantly housed in lymphoid organs, are CD56^bright^, and although less mature (“unlicensed”) (3, 6, 7), they have a greater capability to secrete and respond to cytokines (8, 9). CD56^bright^ and CD56^dim^ NK cells are also distinguished by their differential expression of FcγRIII (CD16), an integral determinant of NK-mediated antibody-dependent cellular cytotoxicity (ADCC), with CD56^dim^ NK cells expressing high levels of the receptor, while CD56^bright^ NK cells are CD16 dim or negative (6). In contrast to T and B lymphocytes, NK cells do not express rearranged, antigen-specific receptors; rather, NK effector function is dictated by the integration of signals received through germ-line-encoded receptors that can recognize ligands on their cellular targets. Functionally, NK cell receptors are classified as activating or inhibitory. NK cell function, including cytotoxicity and cytokine release, is governed by a balance between signals received from inhibitory receptors, notably the killer Ig-like receptors (KIRs) and the heterodimeric C-type lectin receptor (NKG2A), and activating receptors, in particular the natural cytotoxicity receptors (NCRs) NKp46, NKp30, NKp44, and the C-type lectin-like activating immunoreceptor NKG2D (9).

The inhibitory KIRs (iKIRs) with known HLA ligands include KIR2DL2 and KIR2DL3, which recognize the HLA-C group 1-related alleles characterized by an asparagine residue at position 80 of the α-1 helix (HLA-CAsn80); KIR2DL1, which recognizes the HLA-C group 2-related alleles characterized by a lysine residue at position 80 (HLA-CLys80); and KIR3DL1, which recognizes the HLA-Bw4 alleles (9, 10). NK cells also express several activating receptors that are potentially specific for self-molecules. KIR2DS1 has been shown to interact with group 2 HLA-C molecules (HLA-C2), while KIR2DS2 was recently shown to recognize HLA-A*11 (10, 11). Hence, these receptors require mechanisms to prevent inadvertent activation against normal tissues, processes referred to as “tolerance to self.” Engagement of iKIR receptors by HLA class I leads to signals that block NK-cell triggering during effector responses. These receptors explain the “missing self” hypothesis, which postulates that NK cells survey tissues for normal levels of the ubiquitously expressed MHC class I molecules (12, 13). Upon cellular transformation or viral infection, surface MHC class I expression on the cell surface is often reduced or lost to evade recognition by antitumor T cells. When a mature NK cell encounters transformed cells lacking MHC class I, their inhibitory receptors are not engaged, and the unsuppressed activating signals, in turn, can trigger cytokine secretion and targeted attack of the virus-infected or transformed cell (13, 14). In parallel, cellular stress and DNA damage (occurring in cells during viral or malignant transformation) results in upregulation of “stress ligands” that can be recognized by activating NK receptors. Thus, human tumor cells that have lost self-MHC class I expression or bear “altered-self” stress-inducible proteins are ideal targets for NK recognition and killing (14–16). NK cells directly kill tumor cells through several mechanisms, including release of cytoplasmic granules containing perforin and granzyme (16–18), expression of tumor necrosis factor (TNF) family members, such as FasL or TNF-related apoptosis-inducing ligand (TRAIL), which induce tumor cell apoptosis by interacting with their respective receptors Fas and TRAIL receptor (TRAILR) (16–19) as well as ADCC (9).

## Interaction Between Natural Killer Cells and Other Immune Subsets

Increasing understanding of NK cell biology and their interaction with other cells of the immune system has led to several novel immunotherapeutic approaches as discussed in this review. NK cells produce cytokines that can exert regulatory control of downstream adaptive immune responses by influencing the magnitude of T cell responses, specifically T helper-1 (TH1) function (20). NK cell function, in turn, is regulated by cytokines, such as IL-2, IL-15, IL-12, and IL-18 (21), as well as by interactions with other cell types, such as dendritic cells, macrophages, and mesenchymal stromal cells (10, 22, 23). IL-15 has emerged as a pivotal cytokine required for NK cell development and maintenance. Whereas mice deficient in IL-2 (historically the cytokine of choice to expand and activate NK cells) have normal NK cells, IL-15-deficient mice lack NK cells (24).

Several cytokines are also known to inhibit NK cell activation and function, thus playing a crucial role in tumor escape from NK immune surveillance. Recently, considerable attention has been paid to the inhibitory effects of transforming growth factor-beta (TGF-β) and IL-10 on NK cell cytotoxicity (12, 25, 26). Several groups have shown that secretion of TGF-β by tumor cells results in downregulation of activating receptors, such as NKp30 and NKG2D, with resultant NK dysfunction (25, 26). Similarly, IL-10 production by acute myeloid leukemia (AML) blasts induces upregulation of NKG2A with significant impairment in NK function (3).

## Modulation of Antibody-Dependent Cellular Cytotoxicity

The CD56^dim^ subset of NK cells expresses the Fcγ receptor CD16, through which NK cells mount ADCC, providing opportunities for its modulation to augment NK effector function (27, 28). In fact, a number of clinically approved therapeutic antibodies targeting tumor-associated antigens (such as rituximab or cetuximab) function at least partially through triggering NK cell-mediated ADCC. Several studies using mouse tumor models have established that efficient antibody–Fc receptor (FcR) interactions are essential for the efficacy of monoclonal antibody (mAb) therapy, a mainstay of cancer therapy (28, 29). Based on this premise, Romain et al. successfully engineered the Fc region of the IgG mAb, HuM195 targeting the AML leukemia antigen CD33, by introducing the triple mutation S293D/A330L/I332E (DLE). Using timelapse imaging microscopy in nanowell grids (TIMING, a method of analyzing kinetics of thousands of NK cells and mAb-coated targets), they demonstrated that the DLE-HuM195 antibody increased both the quality and quantity of NK cell-mediated ADCC by recruiting NK cells to participate in cytotoxicity via CD16-mediated signaling. NK cells encountering DLE-HuM195-coated targets induced rapid target cell apoptosis by promoting conjugation to multiple target cells (leading to increased “serial killing” of targets), thus inducing apoptosis in twice the number of targets as the wild-type mAb (27).

Additional approaches under investigation to enhance NK cell-mediated ADCC include antibody engineering and therapeutic combination of antibodies predicted to have synergistic activity. For example, mogamulizumab (an anti-CCR4 mAb recently approved in Japan) is defucosylated to increase binding by FcγRIIIA and thereby enhances ADCC. Mogamulizumab successfully induced ADCC activity against CCR4-positive cell lines and inhibited the growth of EBV-positive NK-cell lymphomas in a murine xenograft model (30). These findings suggest that mogamulizumab may be a therapeutic option against EBV-associated T and NK-lymphoproliferative diseases (30). Obinutuzumab (GA101) is a novel type II glycoengineered mAb against CD20 with increased FcγRIII binding and ADCC activity. In contrast to rituximab, GA101 induces activation of NK cells irrespective of their inhibitory KIR expression, and its activity is not negatively affected by KIR/HLA interactions (31). These data show that modification of the Fc fragment to enhance NK-mediated ADCC can be an effective strategy to augment the efficacy of therapeutic mAbs (31).

Although enhanced NK-mediated ADCC occurs in the presence of certain mAbs, in the case of non-engineered mAbs (such as rituximab), this NK-mediated cytotoxicity is typically still under the jurisdiction of KIR-mediated inhibition. However, ADCC responses can be potentiated *in vitro* in the presence of antibodies that block NK cell inhibitory receptor interaction with MHC class I ligands (32). These include the use of anti-KIR Abs to block the interaction of iKIRs with their cognate HLA class I ligands. To exploit this pathway pharmacologically, a fully humanized anti-KIR mAb 1-7F9 (IPH2101) (33) with the ability to block KIR2DL1/L2/L3 and KIR2DS1/S2 was generated. *In vitro*, anti-KIR mAbs can augment NK cell-mediated lysis of HLA-C-expressing tumor cells, including autologous AML blasts and autologous CD138^+^ multiple myeloma (MM) cells (34). Additionally, in a dose-escalation phase 1 clinical trial in elderly patients with AML, 1-7F9 mAb was reported to be safe and could block KIRs for prolonged periods (35). A recombinant version of this mAb with a stabilized hinge (lirilumab) was recently developed. Lirilumab is a fully humanized IgG4 anti-KIR2DL1, -L2, -L3, -S1, and -S2 mAb. The iKIRs targeted by lirilumab collectively recognize virtually all HLA-C alleles, and the blockade of the three KIR2DLs allows targeting of every patient without the need for prior HLA or KIR typing (33, 34). Furthermore, the combination of an anti-KIR mAb with the immunomodulatory drug lenalidomide was shown to potentiate ADCC and is being tested in a phase 1 clinical trial in patients with MM [NCT01217203 (35)]. A potential concern is related to how inhibitory KIR blockade may impact on the ability of NK cells to discriminate self, healthy cells from abnormal virally infected or cancerous cells. Preliminary *in vitro* data suggest that Ab blockade of iKIRs will preferentially augment the ADCC response, without increasing cytotoxicity against self healthy cells (32). It is reassuring that in the IPH2101 phase 1 studies, no alterations in the expression of major inhibitory or activating NK receptors or frequencies of circulating peripheral lymphocytes were reported, indicating that the Ab does not induce clinically significant targeting of normal cells by NK cells (35). Lin et al. recently reported on the application of an agonistic NK cell-targeted mAb to augment ADCC (36). Following FcR triggering during ADCC, expression of the activation marker CD137 is increased. Agonistic antibodies targeting CD137 have been reported to augment NK-cell function, including degranulation, secretion of IFN-γ, and antitumor cytotoxicity in *in vitro* and *in vivo* preclinical models of tumor (36–39). The combination of the agonistic anti-CD137 antibody with rituximab is currently being evaluated in a phase 1 trial in patients with lymphoma [NCT01307267 (35–37)].

Other factors, such as specific CD16 polymorphisms and NKG2D engagement, can also influence ADCC, with certain polymorphisms (such as FcγRIIIa-V158F polymorphism) resulting in a stronger IgG binding (40). These findings are clinically relevant, as supported by the observation that patients with non-Hodgkin lymphoma (NHL) with the FcγRIIIa-V158F polymorphism experienced improved clinical response to rituximab (41, 42). In summary, several antibody combinations designed to boost ADCC have shown promising results in preclinical and early clinical trials, thus warranting further study of this strategy to enhance NK cell activity against tumor cells.

## Adoptive Transfer of Autologous NK Cells

The early studies of adoptive NK cell therapy focused on enhancing the antitumor activity of endogenous NK cells (43). Initial trials of adoptive NK therapy in the autologous setting involved using CD56 beads to select NK cells from a leukapheresis product and subsequently infusing the bead-selected autologous NK cells into patients (43, 44). Infusions were followed by administration of systemic cytokines (most commonly IL-2) to provide additional *in vivo* stimulation and support their expansion. This strategy met with limited success due to a combination of factors (44). Although cytokine stimulation promoted NK cell activation and resulted in greater cytotoxicity against malignant targets *in vitro*, only limited *in vivo* antitumor activity was observed (43–45). Similar findings were observed when autologous NK cells and systemic IL-2 were given as consolidation treatment to patients with lymphoma who underwent autologous BMT (46). The poor clinical outcomes observed with adoptive transfer of *ex vivo* activated autologous NK cells followed by systemic IL-2 were attributed to three factors: (1) development of severe life-threatening side effects, such as vascular leak syndrome as a result of IL-2 therapy; (2) IL-2-induced expansion of regulatory T cells known to directly inhibit NK cell function and induce activation-induced cell death (47–49); and (3) lack of antitumor effect related to the inhibition of autologous NK cells by self-HLA molecules. Strategies to overcome this autologous “checkpoint,” thus redirecting autologous NK cells to target and kill leukemic blasts are the subject of intense investigation (33–35). These include the use of anti-KIR Abs (such as the aforementioned lirilumab) to block the interaction of inhibitory receptors on the surface of NK cells with their cognate HLA class I ligand.

## Exploiting the Alloreactivity of Allogeneic NK Cells – Adoptive Immunotherapy and Beyond

An alternative strategy is to use allogeneic instead of autologous NK cells, thus taking advantage of the inherent alloreactivity afforded by the “missing self” concept (13). Over the past decade, adoptive transfer of *ex vivo*-activated or -expanded allogeneic NK cells has emerged as a promising immunotherapeutic strategy for cancer (24, 50–52). Allogeneic NK cells are less likely to be subject to the inhibitory response resulting from NK cell recognition of self-MHC molecules as seen with autologous NK cells. A number of studies have shown that infusion of haploidentical NK cells to exploit KIR/HLA alloreactivity is safe and can mediate impressive clinical activity in some patients with AML (50–52). In fact, algorithms have been developed to ensure selection of stem cell donors with the greatest potential for NK cell alloreactivity for allogeneic HSCT (50).

Promising results in the HSCT setting suggest that the application of this strategy in the non-transplant setting may be a plausible option. Miller et al. were among the first to show that adoptive transfer of *ex vivo-*expanded haploidentical NK cells after lymphodepleting chemotherapy is safe, and can result in expansion of NK cells *in vivo* without inducing graft-vs.-host disease (GVHD) (50). In a phase I dose-escalation trial, 43 patients with either hematologic malignancies (poor prognosis AML or Hodgkin lymphoma) or solid tumor (metastatic melanoma or renal cell carcinoma) received up to 2 × 10^7^cells/kg of haploidentical NK cells following either low intensity [low-dose cyclophosphamide (Cy) and methylprednisolone or fludarabine (Flu)] or high intensity regimens (Hi-Cy/Flu). All patients received subcutaneous IL-2 after NK cell infusion. Whereas adoptively infused NK cells persisted only transiently following low intensity regimens, AML patients who received the more intense Hi-Cy/Flu regimen had a marked rise in endogenous IL-15 associated with expansion of donor NK cells and induction of complete remission (CR) in five of 19 very high-risk patients. The superior NK expansion observed after high-dose compared to low-dose chemotherapy was attributed to a combination of factors including prevention of host T cell-mediated rejection and higher levels of cytokines, such as IL-15. These findings provided the first evidence that haploidentical NK cells are safe and can persist and expand *in vivo*, supporting the proof of concept that NK cells may be applied for the treatment of selected malignancies either alone or as an adjunct to HSCT (50).

Another pivotal pilot study, the NKAML trial (Pilot Study of Haploidentical NK Transplantation for AML), reported that infusion of KIR-HLA-mismatched donor NK cells can reduce the risk of relapse in childhood AML (51). Ten pediatric patients with favorable or intermediate risk AML in first CR were enrolled following completion of 4–5 cycles of chemotherapy. All patients received a low-dose conditioning regimen consisting of Cy/Flu prior to infusion of NK cells (median, 29 × 10^6^/kg NK cells) from a haploidentical donor, followed by six doses of IL-2. NK infusions were well tolerated with limited non-hematologic toxicity. All patients had transient engraftment of NK cells for a median of 10 days (range 2–189 days) with significant expansion of KIR-mismatched NK cells. With a median follow-up of 964 days, all patients remained in remission, suggesting that donor-recipient HLA-mismatched NK cells may reduce the risk of relapse in childhood AML (51).

Other strategies currently under investigation include the infusion of KIR-ligand-mismatched haploidentical NK cells as part of the pre-HSCT conditioning regimen (NCT00402558), and NK cell infusion to prevent relapse or as therapy for minimal residual disease in patients after haploidentical HSCT (NCT01386619).

## Adoptive NK Cell Therapy in Solid Malignancies

Natural killer cell-based immunotherapies are also a promising therapeutic option for solid tumors. A number of studies have shown that the presence of intratumoral NK cells correlates with delayed tumor progression and improved outcomes (53–55). However, the successful application of NK cell-based therapies in the solid tumor setting poses a special challenge. In addition to the immune evasion strategies common to hematologic malignancies, such as secretion of immunosuppressive cytokines and downregulation of activating ligands (55–57), additional challenges specific to solid tumors exist; NK cells must not only traffic to sites of disease, but also penetrate the tumor capsule in order to exert their effector function. Furthermore, tumor targets must be inherently susceptible to NK-mediated cytotoxicity (58). Several groups have focused on strategies to alter the tumor microenvironment by targeting myeloid-derived suppressor cells or regulatory T cells (Treg) rather than the tumors themselves (58, 59). In fact, the prospect of combining NK cell-based immunotherapy with approaches to target the immunosuppressive tumor microenvironment or immune checkpoints, such as KIR blockade, is especially relevant to the treatment of solid tumors (55, 58). Several early phase clinical trials have demonstrated the feasibility of adoptive therapy with autologous or allogeneic *ex vivo* activated/expanded NK cells in patients with refractory solid malignancies [NCT01875601 (60)]; however, outside of the post-HSCT setting (namely in neuroblastoma), limited data on the clinical efficacy of NK cells in eradicating solid tumors exist. Currently, several trials are actively recruiting patients with refractory solid tumors for adoptive NK therapy (including NCT01807468, NCT02130869, and NCT0210089).

## The Ideal Manufacturing Strategy for *ex vivo* Activation of NK Cells

Recent approaches to adoptive NK therapy focused on infusion of NK cells that have undergone a process of *ex vivo* cytokine activation and expansion (61). A number of cytokines (IL-2, IL-12, IL-15, IL-18, IL-21, and type I IFNs) have been studied to activate and expand NK cells *ex vivo* (62–65). The most extensively studied cytokine is IL-2 (62, 63). This is not surprising, considering IL-2 was the only cytokine available in clinical grade until recently. Nevertheless, NK cells expanded in the presence of IL-12, IL-15, and IL-18, either alone or in combination, have shown remarkable activity against tumor targets in experimental models and offer an attractive strategy for clinical expansion of NK cells (64, 65). IL-15, in particular, is appealing as it does not stimulate Tregs (65). IL-15 has been tested in preclinical models with promising results; however, very high doses were necessary to observe any meaningful *in vivo* antitumor effects, and toxicity of systemic cytokine administration and cytokine-induced NK-cell apoptosis remained major issues (65). Recently, Miller et al. compared the persistence and *in vivo* efficacy of adoptively infused freshly activated NK cells (FA-NK) and *ex vivo*-expanded NK cells (Ex-NK) in a xenotransplantation model. They showed that *in vivo* NK cell persistence is cytokine dependent, with IL-15 being superior to IL-2. They also reported that cryopreservation of FA-NK or Ex-NK was detrimental to NK cell function, and that culture conditions influence homing, persistence, and expansion of NK cells *in vivo* (66).

Although the results from the abovementioned trials proved that transient persistence of adoptively transferred NK cells obtained via apheresis is feasible and safe, the requirement of a willing, available donor precludes the widespread applicability of this approach. Hence, more recent efforts have focused on optimizing methods for *ex vivo* expansion of NK cells from peripheral blood mononuclear cells (PBMCs) collected by a simple blood draw, with a goal of producing large quantities of purified, functionally active NK cells for clinical use. These expansion strategies include the use of “feeder cells,” such as monocytes in the form of irradiated PBMCs, EBV-transformed lymphoblastoid cell lines (EBV-LCLs) or gene-modified, irradiated K562 cells expressing membrane-bound IL-15 or IL-21 and 41BB ligand for costimulation (61, 66–69) in gas-permeable large-scale expansion flasks. These techniques have dramatically increased the yield and activation status of NK cells, potentially overcoming the need for leukapheresis. Because the feeder cells used in these manufacture methods are lethally irradiated prior to use in culture (leaving the remaining feeder cells to be lysed by the expanding NK cells), the risk of infusing viable feeder cells is negligible. However, a number of safeguards have also been incorporated that include monitoring the growth rate of feeder cells and testing for the presence of viable feeder cells at the end of the culture period. Clinical products are, therefore, only released if no viable gene-modified K562 cells or transformed LCLs are present, with strict cutoff values for contaminating B cells and monocytes at the end of the culture period as well (67).

Although these expansion methods can produce large numbers of functionally active NK cells, concomitant expansion of competing cells with immunosuppressive properties, such as Tregs remains problematic. Early studies reported that NK cell infusions from haploidentical donors are able to induce remissions in some patients with AML, but not others (50–52). Several groups, therefore, set out to explore factors that may contribute to the failure of NK expansion *in vivo*. Bachanova explored the effect of competition between Tregs and NK cells in 57 patients with refractory AML who received lymphodepleting chemotherapy followed by NK cell infusion and IL-2 administration [NCT00274846 and NCT01106950 (70)]. Fifteen patients also received the IL-2-diphtheria toxin fusion protein (IL2DT) to deplete Tregs prior to NK cell infusion. IL2DT treatment was associated with increased donor NK cell persistence and improved CR and disease-free survival at 6 months (33 vs. 5% in patients not receiving IL2DT; *P* < 0.01). In the IL2DT cohort, NK cell expansion correlated with higher post-chemotherapy serum IL-15 levels (*P* = 0.002) and effective peripheral blood (PB) Treg depletion (<5%) at day 7 (*P* < 0.01). This study shed light on the importance of optimizing the cytokine milieu to facilitate the *in vivo* expansion of adoptively transferred NK cells and identifying ways to abrogate the immunosuppressive elements, such as regulatory T cells.

Although these data are encouraging, adoptive transfer of NK cells under good manufacturing practices (GMP) requires significant infrastructure and specialized processing equipment, thus limiting the availability and scalability of these NK cell therapies to a few specialized institutions (61). Nonetheless, the feasibility of centralized processing and safe delivery of *ex vivo*-manufactured NK cells for infusion at remote clinics have been demonstrated, suggesting that the practice might become more widespread as procedures are optimized (71). For example, in order to improve access to *ex vivo* activated NK cells and ease the burden associated with producing cellular products at individual treatment centers, the National Heart, Lung, and Blood Institute (NHLBI, Bethesda, MD, USA) sponsored the Production Assistance for Cellular Therapies (PACT) program. Using this approach, activated NK cells have been sent to other centers for infusion into patients (72, 73).

Since the initial reports of successful adoptive transfer of NK cells (50–52), many groups continue to perform extensive preclinical exploration of the ideal manufacturing strategy for *ex vivo* activation and expansion of NK cells. Several expansion methods optimized in the preclinical setting have been successfully scaled up for the clinic (61, 67–70). In addition to the six clinical trials of adoptive NK cell therapy for leukemia that have reported their data (48–52, 70), there are currently 12 active clinical trials enrolling patients with hematologic malignancies for NK cell adoptive therapy, a number that is steadily rising.

## Alternative Sources of NK Cells for Adoptive Transfer – Is Cord Blood the Answer?

Although the majority of clinical studies of NK cell immunotherapy have used PB NK cells, several alternative sources of NK cells exist. These include bone marrow, human embryonic stem cells (hESCs), induced pluripotent stem cells [iPCSs (74, 75)], and umbilical cord blood (CB). While the generation of NK cells from hESCs or iPCS has been largely experimental to date, clinical-grade generation and expansion of NK cells from CB-derived CD34^+^ cells has been successfully achieved (76).

Umbilical CB as a source for NK cells lends additional clinical advantages. CB contains a high percentage of NK cells (77, 78) and serves as an immediate “off-the-shelf” source of NK cells, with less stringent requirements for HLA matching, and lower risk of causing GVHD following infusion due to the naivety of the cord T cell repertoire (77, 78). Although no direct comparison of PB- and CB-derived NKs has been performed in the clinical setting, *in vitro* studies have identified a number of differences between CB and PB NK cells. CB NK cells form weaker conjugates with target cells due to the lower membrane expression of adhesion molecules on their surface (79, 80). CB NK cells also express higher levels of lectin-like inhibitory receptors (CD94/NKG2A) and lower levels of KIRs, indicating an immature phenotype (81). CB NK cells are similarly sensitive to cytokines for *in vivo* expansion and persistence (82). However, it appears that the requirements for *in vitro* expansion of CB NK cells may be different to those required for PB NKs. CB NKs are less responsive to IL-2 stimulation, which may be related to the lower expression of IL-2Rα and reduced activation of the STAT5 signaling pathway as compared with PB NK cells (83). The combination of IL-15 and IL-18, however, can induce significant proliferation and cytokine production by CB NK cells, while the killing capacity of CB NK cells is significantly enhanced after stimulation with IL-15 (83). As with PB-derived NK cells, T-cell contamination is a concern, but can be ameliorated by CD3 depletion. T-cell contamination should be limited to <1–5 × 10^5^/kg (61) to minimize the risk of GVHD. In addition, CD56^+^ selection reduces B-cell contamination to <1%, which minimizes passenger B lymphocyte-mediated complications, such as EBV-related post-transplant lymphoproliferative disorder (PTLD) and acute hemolytic anemia.

More recently, efforts have focused on optimizing the large-scale expansion of purified CB-derived NK cells. Shah et al. were the first to describe a strategy for expanding NK cells from cryopreserved CB units in which they employed K562-based artificial antigen-presenting cells (aAPCs) expressing membrane-bound IL-21 (clone 9.mbIL21) (77, 84). The clone 9.mbIL21 cell line is GMP-grade and expresses membrane-bound IL-21, 4-1BB ligand, CD64 (FcγRI), and CD86. After only 14 days of culture in a gas-permeable culture system, mean-fold expansion of CB-NK cells was 1848-fold from fresh and 2389-fold from cryopreserved CB with >95% purity for CD56^+^CD3^−^ NK cells. aAPC-expanded CB-NK cells displayed a phenotype similar to that of expanded PB-NK cells and maintained strong expression of the transcription factors eomesodermin and T-bet. Furthermore, CB-NK cells formed functional immune synapses and efficiently killed various MM targets *in vitro*. Finally, aAPC-expanded CB-NK cells showed significant *in vivo* activity against MM in a xenogenic mouse model. These findings highlight a clinically applicable strategy for the generation of highly functional CB-NK cells using an aAPC platform, which can be potentially extended to other hematologic malignancies and solid tumors (77). A number of phase I/II clinical trials are underway to test the feasibility and efficacy of CB-NK cell adoptive therapy in patients with hematologic malignancies (NCT01619761, NCT01729091 NCT02280525, NCT01914263, and NCT00412360) (summarized in Table 1).

**Table 1:** **Published results of NK adoptive immunotherapy trials in hematologic malignancies**.

Reference	Approach	Disease	NK source of cells	Conditioning regimen	Dose of cells	Outcome
Burns et al. (43)	*Ex vivo* IL-2 activated autologous NKs or bolus IL-2	Relapsed lymphoma *N* = 29	Autologous	None	4 × 10^7^–8 × 10^7^ cells/kg	1° endpoint safety/feasibility; no change in outcome compared to historical controls
^a^Miller et al. (50)	IL-2 activated NK cells	HR AML (adults) *N* = 19	Haplo-related donors	Hi-Cy/Flu	1 × 10^6^–2 × 10^7^ cells/kg followed by 14 days IL-2	5/19 (26%) CR
^a^Rubnitz and Inaba (51)	Fresh-activated NK (FA-NK)	LR/IR AML (pedi) *N* = 10	Haplo-related donors	Hi-Cy/Flu	Median 29 × 10^6^ cells/kg followed by IL-2 × 6 doses	10/10 (100%) CR at 964 days
Yoon et al. (49)	IL-7/15/21 *ex vivo* cultured NKs	HR ALL/AML/MDS (adults) *N* = 14	Haplo-related HSCT donors (from CD34^+^ fraction)	Pre-SCT conditioning regimen (Bu/Flu/thymo)	Median 2.2 × 10^6^ cells/kg	1° endpoint safety/feasibility; (no toxicity; low-grade GVHD); 4/14 (28%) alive and well
^a^Curti and Ruggeri (52)	CD56^+^ selected NKs	AML-CR and relapsed (adult) *N* = 13	Haplo-related donors	Hi-Cy/Flu	5 × 10^6^ cells/kg followed by IL-2 × 6 doses	6/13 (46%) remain in CR
Stern et al. (48)	1–3 doses positively selected NKs	ALL, AML (adult and pedi) *N* = 15	Haplo donors	Pre-SCT conditioning regimen	Median 1.2 × 10^7^ cells/kg	4/16 (25%) alive
Klingemann and Grodman (71)	Apheresis-mobilized CD56 selection	HL, NHL, MM *N* = 13	Haplo donors	None	1 × 10^5^–2 × 10^7^ cells/kg	1° endpoint safety/feasibility; 7/13 in remission
^a^Bachanova (70)	NK infusion w/IL-2 ± IL2DT Treg depletion	AML *N* = 42 (IL-2 alone) *N* = 15 (+IL2DT)	Haplo donors	Hi-Cy/Flu	Mean 2.6 ± 1.5 × 10^7^ cells/kg	IL-2 alone: 9/42 (21%) CR/CRi IL2DT: 8/15 CR/CRi (53%)
Choi et al. (116)	Apheresis-mobilized, *ex vivo* IL-15/21 induced NK cells	*N* = 41	Haplo donors	Bu/Flu/ATG	Median 1 × 10^8^ cells/kg	Reduced leukemia progression 46 vs. 74%

*HR, high risk; haplo, haploidentical; LR, low risk; IR, intermediate risk; Hi-Cy/Flu, high-dose cyclophosphamide and fludarabine; CR, complete remission; ALL, acute lymphoblastic leukemia; AML, acute myeloid leukemia; MDS, myelodysplastic syndromes; SCT, hematopoietic stem cell transplant; HL, Hodgkin lymphoma; NHL, non-Hodgkin lymphoma; pedi, pediatric; CRi, complete remission with incomplete platelet recovery; IL2DT, IL-2-diphtheria fusion protein*.

*^a^NK cells infused outside of the setting of hematopoietic cell transplantation*.

## Human NK Cell Lines as a Source of NK Immunotherapy

The adoptive transfer of NK cell lines has several theoretical advantages over the use of patient- or donor-derived NK cells. These are primarily related to the lack of expression of iKIRs, presumed lack of immunogenicity, ease of expansion and availability as an “off-the-shelf” product (85). Several human NK cell lines, such as NK-92 and KHYG-1, have been documented to exert antitumor activity in both preclinical and clinical settings (86–88). NK-92, the most extensively characterized NK-cell line, was established in 1994 from the PB of a male Caucasian patient with NHL. NK-92 cells are IL-2-dependent, harbor a CD2^+^CD56^+^CD57^+^ phenotype and exert potent *in vitro* cytotoxicity (86). Infusion of up to 10^10^ cells/m^2^ NK-92 cells into patients with advanced lung cancer and other advanced malignancies was well tolerated and the cells persisted for a minimum of 48 h with encouraging clinical responses (86, 88–91). However, potential limitations of using NK cell lines, such as NK-92 cells, include the requirement for irradiation to reduce the risk of engrafting cells with potential *in vivo* tumorigenicity, and the need for pre-infusion conditioning to avoid host rejection. Furthermore, infusion of allogeneic NK cell lines may induce T and B cell alloimmune responses, limiting their *in vivo* persistence and precluding multiple infusions. A number of studies are testing NK-92 cells (Neukoplast^®^) in patients with solid tumors, such as Merkel cell cancer and renal cell carcinoma, as well as in hematological malignancies (85).

While results from clinical studies of NK cell adoptive therapy are encouraging (48–52, 70), significant gaps remain in our understanding of the optimal conditions for NK cell infusion. Based on the pioneering work from Rosenberg et al. demonstrating the importance of lymphodepletion to support the expansion of tumor-infiltrating T cells (92) and given its emergence as a key determinant of efficacy with CAR therapy, several groups are actively investigating the ideal preparative regimen to promote the expansion and persistence of adoptively infused NK cells (53, 69, 70, 75). Available data support the use of high-dose Cy/Flu regimen as the frontrunner, considering it is reasonably well tolerated and shown to support the *in vivo* expansion of NK cells (51, 70). IL-15 is an ideal candidate cytokine for the expansion of NK cells *in vivo*, especially since it does not promote expansion of regulatory T cells (66), which have been shown to suppress NK cell effector function in IL-2-based trials (69, 70). In a recent phase 1 study in patients with metastatic melanoma or renal cell carcinoma, rhIL-15 was shown to activate NK cells, monocytes, γδ, and CD8 T cells (93). However, as an intravenous bolus dose, rhIL-15 proved too difficult to administer because of significant clinical toxicities (93). Based on these promising data, alternative dosing strategies are being investigated, including continuous intravenous infusions. To this effect, systemic IL-15 along with infusion of donor NK cells are currently being tested in a phase I clinical trial for AML (NCT01385423).

## Chimeric Antigen Receptor-Modified NK Cells

Chimeric antigen receptors have been used extensively to redirect the specificity of T cells against leukemia (94). Recently, use of an anti-CD19-BB-ζ receptor transduced into autologous or allogeneic T cells produced dramatic clinical responses in patients with acute lymphoblastic leukemia (95, 96); however, infusions of activated T cells from an allogeneic source are likely to increase the risk of GVHD. T cell-depleted allogeneic NK cells, by contrast, should not cause GVHD, as predicted by observations in murine models, as well as in patients with leukemia and solid malignancies treated with haploidentical NK cells (50–52). Given their shorter lifespan and potent cytolytic function, mature NK cells provide attractive candidate effector cells to express CARs and, provide an excellent source of off-the-shelf cellular therapy for patients with cancer.

The feasibility of genetically engineering NK cells to express CARs has been shown in the preclinical setting (97, 98). Primary human NK cells, as well as NK-92 cells, have been successfully engineered to express CARs against a number of targets including CD19, CD20, CD244, and HER2 (97). CAR-transduced NK cells mediate efficient *in vitro* and *in vivo* killing of tumor targets (97, 98) although to date, no clinical data of CAR NK cell therapy have been reported. Shimasaki et al. recently tested the expression of a receptor containing CD3ζ and 4-1BB signaling molecules (anti-CD19-BB-ζ) in human NK cells after mRNA electroporation using a clinical-grade electroporator. The authors reported adequate transfection efficiency 24 h after electroporation, with median anti-CD19-BB-ζ expression of 40.3% in freshly purified and 61.3% in expanded NK cells. NK cells expressing anti-CD19-BB-ζ secreted IFN-γ in response to CD19-positive target cells. Interestingly, the levels of CAR expression in NK cells after mRNA transfection were comparable to those achieved by retroviral transduction. A large-scale protocol was developed to transfect expanded NK cells, achieving excellent receptor expression and considerable cytotoxicity of CAR-transduced NK cells in xenograft models of B-cell leukemia (99). Another interesting strategy is the development of CAR-modified NK cells that target NKG2D ligands on the surface of tumor cells, rendering NK more cytotoxic against a variety of hematologic and solid malignancies (100). NK cells have also been successfully engineered to target antigens on a variety of solid tumors. For example, an NK-CAR targeting the ganglioside GD2 (present on neuroblastoma cells) has been tested in preclinical studies (101, 102). GD2 is also expressed on breast cancer stem cells, thus raising the potential for its widespread use as a target for immunotherapy (103). Additional antigens targeted by NK CARs include HER2 (overexpressed in a number of solid tumors), CD138, and CS1 (overexpressed in MM) (104, 105).

Although these data support the use of CAR engineering to redirect the specificity of NK cells to augment their cytotoxicity, a number of challenges remain. These include the relative difficulty in expressing exogenous genes in primary human NK cells and the need to expand NK cells in culture to achieve adequate numbers for clinical studies of immunotherapy. To counteract this difficulty, some groups have expressed CARs in the human NK-like cell line NK-92, in an attempt to engineer a uniformly cytolytic effector cell population (106). As previously mentioned, NK-92 cells can be easily expanded in culture and their safety has been shown in phase I clinical trials in human subjects. Thus, CAR-expressing NK-92 cells may offer a practical source of cells for NK cell-based immunotherapeutic trials. In order to prevent the risk of engrafting cells with potential *in vivo* tumorigenicity, however, NK-92 cells must be irradiated prior to infusion, which may in turn significantly impact their *in vivo* persistence and long-term antitumor efficacy. Although limited *in vivo* persistence could prove beneficial once the alloreactive NK cells have eradicated the tumors, a number of studies of adoptive therapy with NK cells and CAR-modified T cells have reported the importance of cell persistence in inducing long-term antitumor response (50, 95, 96).

As with CAR-modified T cell therapy, a number of variables can affect the activation, antitumor efficacy, and persistence of CAR-NK cells. Second and third generation CAR constructs incorporating additional costimulatory domains (e.g., CD28, OX-40, or 4-1BB) have been shown to enhance both *in vitro* and *in vivo* activation, and the persistence of CAR T cells. Further studies exploring the optimal vector, construct and transduction method are necessary to identify the “perfect NK CAR.”

## Safety Concerns Related to Adoptive Transfer of Car-Modified NK Cells

When considering the use of CAR-modified effector cells, one must take into account their safety profile. Many of the same concerns raised with CAR-modified T cells may be relevant to CAR-NK cells. These include on-target/off-tumor effects, GVHD, cytokine release syndrome, tumor lysis syndrome, and toxicity to normal tissues due to limited selectivity of the target antigen (107–109). Thus, the necessity of equipping CAR-modified NK cells with a “safety switch” or suicide gene is an important question to explore. While mature allogeneic CAR-engineered NK cells are expected to be short lived, data on the persistence of more immature NK cells, such as those derived from CB, are lacking. Interestingly, a recent study reported that IL15/4-1BBL-activated NK cells infused early after T-depleted allogeneic stem cell transplantation in patients not receiving immunosuppressive prophylaxis could contribute to acute GVHD (110). To this effect, the insertion of a suicide safety switch system, as employed with CAR-modified T cells (111, 112), can provide an efficient means for depletion of these cells if needed. Inducible suicide systems have safely and effectively eradicated GVHD in patients receiving adoptively transferred T cells without causing deleterious effects (112). However, these systems have not been extensively studied in NK cells, and in the absence of clinical data on the *in vivo* persistence of CAR-modified NK cells, the necessity of a suicide switch in this setting remains unknown.

Despite the growing wealth of preclinical experience with CAR-engineered NK cells, to date, only two clinical studies (both targeting CD19^+^ malignancies using a retroviral transduced anti-CD19-BB-ζ NK-CAR) have obtained regulatory approval: one is a recently completed pediatric study at St. Jude Children’s Research Hospital, where haploidentical NK cells modified with anti-CD19-BB-ζ CAR were infused into patients with B-ALL (ClinicalTrials.gov.NCT00995137) and the other is an ongoing study at the National University Hospital in Singapore (ClinicalTrials.gov.NCT01974479) using IL-2-activated haploidentical CAR-modified NK cells in pediatric and adult patients with refractory B-ALL (99). The results of these studies have not been reported to date.

## Bispecific and Trispecific Engagers

An innovative immunoglobulin-based strategy to redirect NK cytotoxicity towards tumor cells is to create either bispecific or trispecific antibodies (BiKE, TriKE) (113). BiKEs are constructed by joining a single-chain Fv against CD16 and a single-chain Fv against a tumor-associated antigen (BiKE), or two tumor-associated antigens (TriKE). Gleason et al. showed that bispecific (bscFv) CD16/CD19 and trispecific (tscFv) CD16/CD19/CD22 engagers directly trigger NK cell activation through CD16, significantly increasing NK cell cytolytic activity and cytokine production against various CD19-expressing B cell lines. The same group also developed and tested a CD16 × 33 BiKE in refractory AML and demonstrated that the potent killing by NK cells could overcome the inhibitory effect of KIR signaling (113, 114).

Notably, activated NK cells lose CD16 (FcRγIII) and CD62L through a metalloprotease called ADAM17, which is expressed on NK cells, which may in turn impact on the efficacy of Fc-mediated cytotoxicity (115). Romee et al. recently showed that selective inhibition of ADAM17 enhances CD16-mediated NK cell function by preserving CD16 on the NK cell surface, thus enhancing ADCC (115). Additionally, Fc-induced production of cytokines by NK cells exposed to rituximab-coated B cell targets can be further enhanced by ADAM17 inhibition. These findings support a role for targeting ADAM17 to prevent CD16 shedding and to improve the efficacy of therapeutic mAbs. The same group subsequently discovered that ADAM17 inhibition enhances CD16 × 33 BiKE responses against primary AML targets (114).

## NK Cells – What Does the Future Hold?

**Figure 1:**
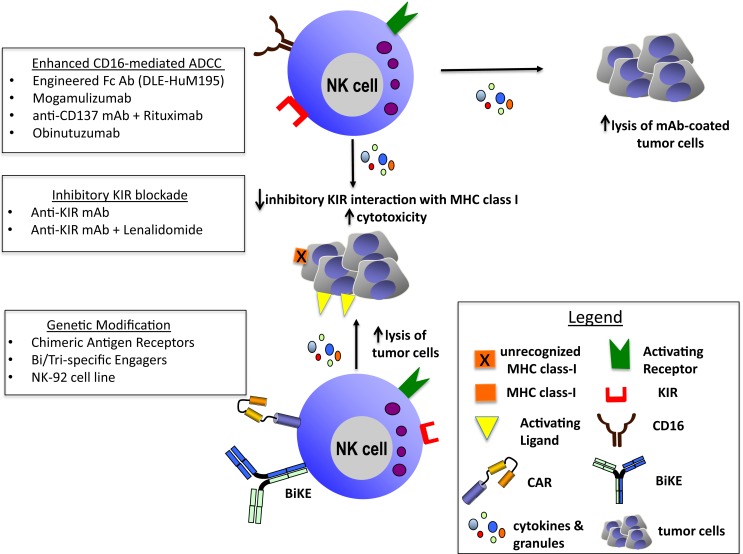
**Enhanced CD16-mediated ADCC: engineered Fc Ab (DLE-HuM195) (27), mogamulizumab (30), anti-CD137 mAb + rituximab (36–39), and obinutuzumab (31). Inhibitory KIR blockade: anti-KIRmAb (33–35) and anti-KIR mAb + lenalidomide (35). Genetic modification: chimeric antigen receptors (97–105), bi/trispecific engagers (113, 114), and NK-92 cell line (86–91)**.

Recent advances in the understanding of NK cell immunobiology have paved the way for novel and innovative anti-cancer therapies. Here, we have discussed a representation of these novel immunotherapeutic strategies to potentiate NK cell function and enhance antitumor activity including ADCC-inducing mAbs, *ex vivo* activated or genetically modified NK cells and bi- or trispecific engagers (Figure 1).

Although experience has shown that adoptive immunotherapy with allogeneic NK cells may be more efficacious than with autologous NK cells, to date, their long-term antitumor benefits have been modest (3). Expansion and persistence of NK cells following infusion appear to be the main determinants of clinical response (50–52, 70), thus underscoring the importance of identifying ways to enhance their persistence and antitumor activity. It is likely that the combination of high-dose lymphodepleting chemotherapy with additional modifications (such as Treg depletion, *in vivo* administration of cytokines, such as IL-15 or enhancement of CD16-mediated antigen targeting) may maximize NK persistence and efficacy.

In addition, the possibility of third-party “off-the-shelf” products with partially HLA-matched NK cells from CB, third-party donors, or NK cell lines allow the advantage of unlimited sources of cells to improve the practicality of cell therapy. With increasing focus on genetically modifying NK cells to redirect their specificity or engager-modified NK cells, it is likely that NK cells will move to the forefront of cancer therapy over the next few years.

## Author Contributions

RR and KR wrote and edited the manuscript.

## Conflict of Interest Statement

The authors declare that the research was conducted in the absence of any commercial or financial relationships that could be construed as a potential conflict of interest.
